# Adipokines as Regulators of Autophagy in Obesity-Linked Cancer

**DOI:** 10.3390/cells11203230

**Published:** 2022-10-14

**Authors:** Alin García-Miranda, Alejandra Garcia-Hernandez, Eduardo Castañeda-Saucedo, Napoleon Navarro-Tito, Paola Maycotte

**Affiliations:** 1 Laboratorio de Biología Celular del Cáncer, Facultad de Ciencias Químico Biológicas, Universidad Autónoma de Guerrero, Chilpancingo 39090, Mexico; 2 Laboratorio Estatal de Salud Pública, Secretaría de Salud, Chilpancingo 39715, Mexico; 3 Centro de Investigación Biomédica de Oriente, Instituto Mexicano del Seguro Social, Puebla 74360, Mexico

**Keywords:** adipokines, autophagy, cancer, obesity, leptin, adiponectin, resistin, apelin, visfatin

## Abstract

Excess body weight and obesity have become significant risk factors for cancer development. During obesity, adipose tissue alters its biological function, deregulating the secretion of bioactive factors such as hormones, cytokines, and adipokines that promote an inflammatory microenvironment conducive to carcinogenesis and tumor progression. Adipokines regulate tumor processes such as apoptosis, proliferation, migration, angiogenesis, and invasion. Additionally, it has been found that they can modulate autophagy, a process implicated in tumor suppression in healthy tissue and cancer progression in established tumors. Since the tumor-promoting role of autophagy has been well described, the process has been suggested as a therapeutic target in cancer. However, the effects of targeting autophagy might depend on the tumor type and microenvironmental conditions, where circulating adipokines could influence the role of autophagy in cancer. Here, we review recent evidence related to the role of adipokines in cancer cell autophagy in an effort to understand the tumor response in the context of obesity under the assumption of an autophagy-targeting treatment.

## 1. Introduction

The global prevalence of obesity has tripled in the last half-century. In 2016, 39% of the adult population (>18) was overweight, 13% was obese, and the prevalence of both conditions was more frequent in females [1,2]. The leading cause of excess body weight and obesity is the energy imbalance between calories consumed and calories expended. The intake of foods rich in fats and sugars has increased, while physical activity has decreased, which is a consequence of environmental and social changes associated with urban development [2,3]. Excess body weight and obesity substantially increase the risk of developing metabolic, cardiovascular diseases, and cancer. Regarding cancer, the risk of developing endometrial, esophageal, colon and rectum, liver, pancreas, kidney, and postmenopausal breast cancer is increased in individuals with these conditions [3]. Biological mechanisms linking obesity to cancer involve the adipose tissue, mainly composed of adipocytes, endothelial cells, fibroblasts, and immune cells [4]. Particularly, the remodeling and expansion of visceral adipose tissue leads to the increased secretion of hormones, adipokines, inflammatory cytokines, growth factors, enzymes, and free fatty acids that, in a paracrine manner, contribute to cancer initiation and progression [5]. The circulating levels of various adipokines have been proposed as prognostic markers for obesity-associated cancers [6,7,8]. While in vitro and in vivo models have described the molecular and cellular mechanisms by which adipokines affect tumor cells [9], recent evidence has been generated suggesting a regulatory role of adipokines in a cellular degradation process called autophagy. Autophagy has been associated with tumor progression, so its potential as a therapeutic target has been suggested [10]. However, the effect of autophagy inhibition in cancer is highly dependent on the tumor context. Interestingly, little has been determined on the role of adipokines in cancer cell autophagy and their implications for tumor progression. Therefore, in this review, we briefly describe the role of adipokines, their regulation of autophagy in cancer, and possible outcomes for obese patients in response to an autophagy-targeted treatment.

## 2. Obesity, Adipose Tissue, and Adipokines in Cancer

Over the last decade, the prevalence of obesity has increased exponentially worldwide, with the United States, Mexico, and New Zealand leading the statistics [1]. Overweight and obesity occur when there is an excessive or abnormal accumulation of fat, and body mass index (BMI) is the most common indicator used to identify these conditions in adults [2]. The BMI is calculated by dividing a person’s weight in kilograms by the square of their height in meters. An individual is considered as overweight when his/her BMI is between 25 and 29.9 kg/m^2^, and obese when the BMI exceeds 30 kg/m^2^ [2]. Both overweight and obesity contribute to the development of metabolic, cardiovascular pathologies, and cancer due to changes in the secretory and metabolic functions experienced by the expanding adipose tissue that impact other tissues and cell types [11]. Of total body fat, visceral adipose tissue is the most biologically active. So, somatometric parameters considered as surrogates of visceral adiposity, such as waist circumference (WC), which is considered to be more strongly associated with visceral fat than BMI, has been related to an increased risk of cardiovascular disease and type 2 diabetes [12]. In cancer, the evidence is controversial, since some studies have related WC (or waist hip ratio, WHR) to increased risk of colorectal and postmenopausal breast cancer, but this association has not been replicated in subsequent reports. For different cancer sites, studies have described similar risk estimates in pancreatic, endometrium and ovarian cancers for BMI and WC [3,12].

**Figure 1:**
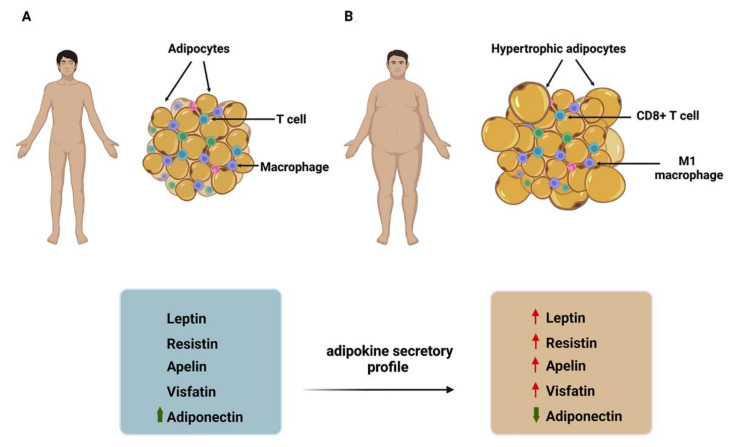
Changes in adipose tissue during obesity. (**A**) Adipose tissue of normal-weight individuals, (**B**) changes in adipose tissue occurring in obesity. During obesity, adipose tissue expansion occurs, characterized mainly by adipocyte hypertrophy. At the cellular level, a change in the secretory profile of adipokines has been reported, highlighting a decrease in adiponectin and an increase in leptin, resistin, apelin, and visfatin. The four short isoforms contain a conserved box1 motif involved in JAK2 recruitment.

Expanding adipose tissue consists mainly of hypertrophic adipocytes, endothelial cells, fibroblasts, a fraction of M1 macrophages, and CD8^+^ T cells (Figure 1) [4]. In this context, the altered secretion of adipokines and inflammatory cytokines from expanding adipose tissue is one of the mechanisms explaining the link between obesity and cancer (Figure 1) [11], where adipocytes and immune cells contribute to the proinflammatory secretory profile of cancer-associated adipose tissue. For example, immune cells are responsible for the high production of pro-inflammatory cytokines such as TNF-α and IL-6 [13]. In contrast, adipocytes secrete mainly adipokines, stimulating macrophages to produce more adipokines or pro-inflammatory cytokines [14].

About 20 adipokines with distinct functions have been characterized. Among them, leptin, resistin, visfatin and apelin are abundant in the plasma of obese individuals, correlate positively with the amount of fat, and have tumor-promoting functions. In contrast, adiponectin has an anti-tumor role, and its plasma concentrations are minimal in obese individuals [15]. The following section discusses general aspects of the effects of adipokines on the tumor.

### 2.1. Leptin

Leptin is a 16 kDa protein encoded by the *LEP* gene in humans and the *ob* gene in mice [16]. It is mainly produced by white adipose tissue, and its plasma concentration correlates directly with the amount of above-mentioned tissue [17]. In normal-weight individuals, leptin serum concentration fluctuates between 2.65 and 46 ng/mL [18], whereas in obese subjects, it is possible to find leptin concentrations as high as 100 ng/mL [19,20], and even a 150 ng/mL concentration could be reached in cases of morbid obesity [20].

Leptin acts through binding to its receptor LEPR/OBR, belonging to the family of class I cytokine receptors without intrinsic kinase activity [21]. LEPR/OBR has six isoforms, one soluble isoform (LEPRe), four short isoforms (LEPRa, LEPRc, LEPRd, and LEPRf), and a long isoform (LEPRb). All isoforms share a similar extracellular and intramembrane domain, while the intracellular domain is variable [22].

However, the long LEPRb/OBRb isoform is the only one that contains the full-length intracellular domain including tyrosine residues required for full receptor activation and signaling [23,24]. LEPRb/OBRb is primarily expressed in the hypothalamus [25] and to a lesser extent in skeletal muscle, kidney, adipocytes, immune system cells, mammary gland, liver, and pancreas [23]. After leptin binding to its receptor, it promotes the recruitment and autophosphorylation of the Janus kinase 2 (JAK2) that phosphorylates other receptor residues. These phosphorylation events enable the activation of signaling pathways such as mitogen-activated protein kinase (MAPK), signal transducer and activator of transcription 3 (STAT3), and phosphoinositide 3-kinase (PI3K) [21,26]. Leptin signaling is regulated by the suppressor of cytokine signaling 3 (SOCS-3) and protein tyrosine phosphatase 1B (PTP-1B) that dephosphorylates JAK2, thus avoiding the activation of downstream signaling cascades [26].

Among all adipokines, the role of leptin in cancer is the most studied. Leptin has been suggested as a potential biomarker of breast cancer risk and cancer progression in women [27], since it has been found to be overexpressed in breast tumors and metastatic lesions [28,29,30,31]. Its primary pro-tumor mechanism is the activation of canonical signaling pathways mediated by the ObR-b receptor, such as JAK2/STAT3, PI3K/AKT and MAPK/ERK, and by non-canonical pathways such as PKC, JNK, MAPK/p38 and AMPK [28]. JAK2 activation leads to the activation and nuclear translocation of STAT3, which regulates the transcription of proliferation genes such as cyclin D1 [29]. The PI3K/AKT signaling cascade is involved in the expression of epithelial–mesenchymal transition (EMT) markers, including vimentin, E-cadherin, MMP-2, MMP-9, Twist, and β-catenin [30,31], and MAPK/ERK activation promotes the secretion of matrix metalloproteases (MMPs), such as MMP-2 and MMP-9, necessary for extracellular matrix degradation [32]. Leptin also participates in the maintenance and support of cancer stem cells through the STAT3 pathway, either by regulating the lipid metabolism [33] or by increasing the expression of stemness transcription factors NANOG, SOX2, and OCT4 [34]. In the regulation of metabolism, leptin promotes the expression of glycolytic enzymes, such as hexokinase II and AKT-mediated glucose transporters [35]. Additionally, it promotes PKM2 enzyme expression associated with epithelial–mesenchymal transition [36], and promotes fatty acid oxidation and oxidative phosphorylation by regulating the c-Myc/PGC-1 axis [37] or by modulating mitochondrial dynamics and biogenesis [38,39].

### 2.2. Resistin

Most of the current information on resistin has been described in murine models. Murine resistin shares 60% homology with its human homolog [40], but the characterization of the biological functions of human resistin is still in progress. In humans, resistin is a 12 kDa protein member of the resistin-like molecule (RELM) family of cysteine-rich proteins [41]. Its serum concentration in humans varies from 7 to 22 ng/mL [40], and it is detected in trimeric and oligomeric isoforms, the latter being associated with a pro-inflammatory function [41]. The highest contributors to plasma resistin levels are monocytes and macrophages associated with adipose tissue [42]. Currently, the receptor for resistin is unknown, but Toll-like receptor 4 (TLR4) and adenylyl cyclase-associated protein 1 (CAP1) have been proposed as candidates [43]. 

Preclinical and clinical studies have reported high serum resistin levels in patients with various types of cancer, generating an increased mortality rate compared to patients expressing low resistin levels. Interestingly, high resistin levels are not only present in patients with obesity-influenced cancers, but also in patients with obesity-independent cancer [8,44,45,46,47]. It has been proposed that resistin signals through TLR4, which mainly activates MAPK and PI3K/AKT signaling pathways, thereby promoting survival and proliferation, and by activating NFκB, it increases IL-6 secretion, which can act paracrinally and induce EMT and metastasis [48]. However, signaling varies among tumor types, with different cellular effects during tumor progression. In ovarian cancer cells (HO-8910), resistin induced the expression of VEGF through the PI3K/AKT-Sp1 pathway [49]. In human gastric cancer cells, resistin induced SDF-1 expression and promoted angiogenesis [50]; and in osteosarcoma cells, ERK, JNK, and p38 pathways were primarily responsible for inducing VEGF-mediated angiogenesis [51]. In breast cancer, resistin induced the phosphorylation of c-Src, PP2A, PKCα, ezrin, radixin and moesin, and increased vimentin expression, promoting cell invasion and metastasis [46]. Resistin treatment in chondrosarcoma cells also increased cellular invasion and secretion of MMP-2 through the AMPK and p38 pathways, while suppressing miR 519d [52]. In addition, it has been reported that resistin induced cellular proliferation, resistance to chemotherapy, and EMT (decreased E-cadherin and increased ZEB1 and vimentin levels) in ovarian cancer cells [53]. Furthermore, Src/EGFR, NFκB, and PI3K were shown to participate in invasion and cell migration signaling in lung cancer cells after resistin exposure [54].

### 2.3. Visfatin

Visfatin is a 52 kDa protein, a product of the pancreatic beta-cell growth factor (PBEF) gene, synthesized primarily by adipocytes and macrophages of visceral adipose tissue and to a lesser extent by the liver, skeletal muscle, neutrophils, and fetal membranes [55]. Visfatin has an enzymatic activity that is involved in the transformation of Nicotinamide (NAM) into Nicotinamide Mononucleotide (NMN) [56]; its plasma levels correlate positively with obesity [8] and specifically with the visceral adipose tissue [57]. In obese women without additional diseases, visfatin levels of 78.6 ± 44.0 ng/mL have been reported, whereas 50.6 ± 26.0 ng/mL were detected in normal-weight women [58]. In a different study, visfatin levels in the serum of overweight subjects were 4.2 ± 0.9 ng/mL, and 2.0 ± 0.6 ng/mL in normal-weight subjects [59]. In addition to obesity, the presence of hormonal or metabolic conditions can influence visfatin plasma concentrations. In patients with different types of cancer, a meta-analysis revealed visfatin concentrations ranging from 0.14 to 171.8 ng/mL in control subjects, and from 0.35 to 222.2 ng/mL in cancer patients, and despite differences in plasma visfatin levels among the different studies analyzed, higher visfatin levels in cancer patients than controls were found, indicating an association between high visfatin and an increased risk of various cancer types [8]. It has also been suggested as a biomarker for early cancer detection [8]. Visfatin is also known as Nampt and PBEF, and was initially described as an adipokine exhibiting insulin-mimetic activity in a retracted paper [60]. Despite this, some authors suggest that the biological role of visfatin could be mediated by insulin receptors, AKT, and MAPK activation [61]. Visfatin has essential functions as a growth factor, cytokine, and cell cycle regulator [55]. There is currently much debate about its regulatory mechanisms, but it has been observed that visfatin transcription is regulated by pro-inflammatory cytokines, such as TNF-α and IL-6 [62], and hypoxia-inducible factor 1α (HIF-1α) [63]. Both mechanisms have been described in cultures of 3T3-L1 adipocytes. 

Although a specific receptor for visfatin has not been described, it has been shown to induce tumor cell proliferation by promoting cellular survival through NF-κB/Notch1, AKT/GSK-3β/β-catenin, c-Abl/STAT3, AKT/ERK1/2, and acetylated SIRT1/p53, and by promoting the G1 to S transition of the cell cycle [56,64,65,66,67,68,69,70]. Likewise, it promotes cell migration and invasion by inducing the expression of the NF-κB-dependent transcription factor Snai1 [71,72]. Additionally, visfatin increases MMP-2 secretion through the ERK activation-mediated transcription factor AP-1 [67] and the promotion of EMT by enhancing the expression of mesenchymal markers (N-cadherin, ZEB1, and vimentin) [71].

### 2.4. Apelin

Apelin is a peptide described as the ligand of the G protein-coupled APJ receptor [73]. Three active forms of apelin have been identified, consisting of 13, 17, and 36 amino acids and a pyroglutamated apelin-13 (Pyr(1)-apelin-13) that originates from a 77 amino acid pre-peptide common precursor [74]. The latter exhibits high resistance to degradation and is considered to have the highest physiological relevance to the APJ receptor [75]. Apelin is a newly-described adipokine produced and secreted by adipocytes that increases under conditions of obesity, mainly associated with hyperinsulinemia [75]. Although high apelin levels have been found in obesity, clinical studies have reported a wide range of apelin plasma levels in healthy subjects and patients of different pathologies. Thus, diverse factors have been related to the positive regulation of apelin in adipocytes, including insulin, TNFα, the overexpression of PPARγ, and PGC1α [66,76]. Additionally, the expression and secretion of apelin increases under hypoxia, with HIF-1 being the primary mediator [77]. On the other hand, its negative regulators are glucocorticoids, which decrease apelin messenger RNA levels [78].

An initial study described that, when overexpressed in cancer cells, apelin has been associated with vascularization and tumor growth in mice [79]. After this finding, several studies in cancer patients, tumor biopsies and cell lines, reported elevated levels of apelin in circulation at the protein level and at the mRNA level, especially in lung, colon, gastroesophageal, hepatocellular, and breast cancer [47,80,81,82,83]. Thus, the presence of apelin has been implicated in carcinogenesis and is associated with an increased risk for cancer development [84,85]. Additionally, apelin favors tumor progression by promoting proliferation, cell migration, invasion, angiogenesis, and metastasis [86,87,88,89,90]. It has been reported that apelin induces non-squamous cell carcinoma proliferation and cell migration under hypoxic conditions in a HIF-1α-dependent manner [87]. On the other hand, in lung adenocarcinoma, apelin-13 (the 13 amino acid form) induces proliferation through the activation of ERK1/2, facilitating the expression of cyclin D1 and the induction of autophagy [90]. In MCF-7 breast cancer cells, apelin-13 induces the transcription of the hormone-dependent breast cancer amplified 1 (AIB1), which promotes the activation of ERK, favoring the expression of cyclin D1 and the secretion of MMP-1, leading to an increase in cell proliferation and invasion [89]. Additionally, apelin-13 promotes lung adenocarcinoma cell migration via the PAK1-cofilin pathway [88]. Likewise, activation of the APJ receptor leads to the phosphorylation of AKT by the inhibition of adenylate cyclase [82]. The apelin/APJ axis primarily activates the ERK and PI3K/AKT signaling pathways, promoting cancer characteristics such as proliferation, migration, and invasion [47]. It has also been described that apelin is involved in the regulation of some factors involved in metastasis, such as MMP, OPN, BMP-2, focal adhesion kinase (FAK), platelet-derived growth factor (PDGF), and p21-activated kinase (PAK). These effects are mediated by the phosphorylation of cofilin, a disintegrin and metalloproteinase with thrombospondin motifs (ADAMTs) and stromal cell-derived factor 1 chemokine receptor alpha 4 (SDF-1α/CXCR4)-mediated signaling. Finally, apelin also acts on endothelial cells, increasing their proliferation and the development of new blood vessels [76].

### 2.5. Adiponectin

Adiponectin is a 30 kDa monomeric protein reported under different names: Acrp30 [91], AdipoQ [92], apM1 [93] or GBP28. Like leptin, monomeric adiponectin is preferentially synthesized and secreted in white adipose tissue [94]. It is the target of post-translational modifications (e.g., glycosylation and hydroxylation), which determine its activity and receptor binding [95]. Before being secreted, it clusters into oligomeric forms: a low-molecular weight trimer (LMW), a medium-molecular weight hexamer (MMW), and a high-molecular weight multimer (HMW) considered to have the highest biological activity [96]. Even though adiponectin is secreted by adipocytes, in obesity, adiponectin levels correlate inversely with the amount of adipose tissue, so the highest adiponectin levels are detected in normal-weight individuals at concentrations of 2 to 30 µg/mL [97]. This occurs because, in a normal individual, adipose tissue is in balance with all its components, adipocytes, and immune cells. However, in obesity, the expanding adipose tissue promotes an inflammatory profile that alters the microenvironment, and contributes to decreased adiponectin secretion and diminished plasma adiponectin [98]. Adiponectin signaling is mediated by binding to AdipoR1, AdipoR2, and T-cadherin receptors [99]. AdipoR1 is expressed in the liver, skeletal muscle, macrophages, and hypothalamus, whereas AdipoR2 is expressed in the liver, brown adipose tissue, and blood vessels [100]. T-cadherin is expressed in different tissues and cell types including the nervous system, the cardiovascular system, skeletal muscle, retina, pancreas, and epithelia such as the skin, prostate, mammary gland and intestine [101]. Each receptor triggers distinct signaling pathways; AdipoR1 is related to AMPK activation and AdipoR2 to PPARα, while T-cadherin is essential for cell adhesion, cellular interactions, and calcium-mediated cell-to-cell signaling [96,100]. Adiponectin signaling is regulated by the downregulation of its receptors, which is mediated by the PI3K/FOXO pathway [100].

Unlike leptin, apelin, resistin, and visfatin, plasma levels of adiponectin are low in obese individuals, and it is the only adipokine recognized to have antitumor effects [11]. Adiponectin can reduce the development and progression of several malignancies, such as breast, colon, and lung cancer, through different molecular mechanisms mediated by the recruitment of the adaptor protein APPL1 and the activation of AMPK, mTOR, PI3K/AKT, MAPK, STAT3, and NF-kB pathways [96,98]. One of the main mechanisms of action described for adiponectin as an antitumor agent is the inhibitory effect observed on the PI3K/AKT/mTOR axis. For example, in a triple-negative breast cancer cell line, adiponectin inhibits the PI3K/AKT pathway and activates AMPK, which phosphorylates Sp1 and represses cyclin D1 expression, leading to an arrest in cellular proliferation [102]. In addition, the inhibitory effects of adiponectin on the proliferation and invasion of lung cancer cells is mediated by an alteration of cell cycle kinetics by inhibiting CREB [103], reducing the activation of inflammatory pathways through the NF-κB-AdipoR1 pathway, and increasing the levels of anti-inflammatory cytokines such as IL-10 [104].

## 3. Autophagy in Cancer

Autophagy is an intracellular degradation process, characterized by the formation of a double membrane vesicle called the autophagosome, where cytoplasmic content, organelles, and target proteins are engulfed for their degradation after fusion with lysosomes [105]. This degradation mechanism is constitutively active at low levels in all cells, and its primary function is to remove damaged structures that could be toxic for cellular function [106]. Under different cellular stress conditions (energetic, nutritional, hypoxic, or oxidative), autophagy increases and promotes cell adaptation and survival [107]. 

**Figure 2:**
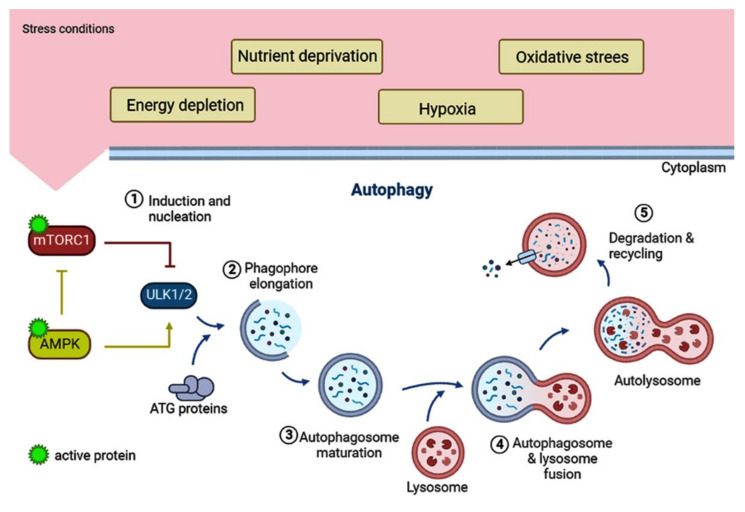
The autophagic process and its regulation. Autophagy is active at low levels in all cells maintaining cytoplasmic homeostasis. However, under different cellular stress conditions such as energy depletion, nutrient deprivation, hypoxia or oxidative stress, autophagy can be induced to higher levels, allowing the cells to survive. The intracellular regulation of autophagy involves the two main kinases sensing growth factors, amino acids, glucose and energy status: AMPK and mTORC1. Under nutrient-rich conditions, mTORC1 phosphorylates and inactivates the ULK1/2 complex, preventing autophagy. Upon nutrient starvation, mTORC1 is inactivated, allowing the induction of autophagy. Alternatively, energy depletion or hypoxia alter the ratio of AMP and ATP, activating AMPK. Active AMPK can inactivate mTORC1 promoting autophagy (1). AMPK can also directly phosphorylate and activate the ULK1/2 complex and induce autophagy (1). After activation of the ULK1/2 complex, other protein complexes necessary to consolidate nucleation are recruited. Several ATG proteins participate from phagophore elongation (2) to autophagosome maturation (3). Next, the autophagosomes fuse with lysosomes (4) for the degradation of their contents. Finally, the autophagolysosomal content is released into the cytoplasm for recycling (5).

Intracellularly, mTORC1 and AMPK kinases are the main regulators of autophagy (Figure 2) [108]. When mTORC1 is active, it keeps the autophagy initiation complex ULK1/2 repressed, and is a negative autophagy regulator [109]. Active AMPK, on the other hand, is a positive regulator that induces autophagy, directly or indirectly. Directly, it functions by activating the ULK1/2 complex, and indirectly by inactivating mTORC1, allowing the release of ULK1/2 and the induction of autophagy [110]. The autophagic process involves a series of coordinated steps involving more than 30 autophagy-related (ATG) proteins that form complexes at each of the five different stages of autophagy (Figure 2): (1) induction and nucleation, (2) expansion or elongation of the phagophore, (3) maturation of the autophagosome, (4) lysosomal fusion, and (5) degradation of cargo elements [109]. Due to the critical role of autophagy in maintaining cellular homeostasis, defects in the autophagic process have been associated with some pathologies such as Alzheimer’s, Parkinson’s, myopathies, and cancer [110].

Autophagy has different functions in cancer: it has a tumor-suppressive function in normal cells and the early stages of carcinogenesis, and a tumor-promoting function in an established cancer [111]. The first evidence for the role of autophagy as an antitumor process comes from studies in murine models in which a monoallelic deletion of beclin-1 was generated, leading to deficient autophagy. Beclin-1 heterozygous-deficient mice presented a high incidence of tumors [112,113] indicating that autophagy functions as a tumor suppressor process in vivo [113]. It is now recognized that the tumor suppressor functions of autophagy involve mechanisms preventing DNA damage and mutation accumulation [111]. For example, the autophagic elimination of aggregated proteins or dysfunctional mitochondria prevents the accumulation of reactive oxygen species (ROS) that are highly genotoxic, thus avoiding oncogenic risk conditions [10]. 

In established tumors, the activation of autophagy by stress conditions (hypoxia, low nutrients and growth factors, oncogenes such as Ras, or inactivation of the p53 tumor suppressor [114]) maintain cancer cell survival and promote tumor growth [115,116]. In this regard, autophagy helps to meet the high metabolic energetic demands of tumor cells that overcome microenvironmental stress and achieve tumor progression [117]. Some autophagy markers have been found increased in metastatic sites compared to primary tumors, and are associated with poor prognosis [118,119,120]. Evidence shows that autophagy participates in the different steps of the metastatic cascade, promoting cancer cell migration and invasion, EMT, and anoikis resistance [121,122]. Likewise, the implementation of cancer treatments such as radiotherapy and chemotherapy activates autophagy, which reduces the likelihood of favorable patient outcomes [123,124,125]. Given the pro-tumoral role of autophagy, it has been proposed as a potential therapeutic target [126]. In clinical trials, the use of hydroxychloroquine, a broad-spectrum inhibitor of autophagy, in association with chemotherapy or radiotherapy, has been applied to patients with different types of solid tumors with controversial results [127,128]. Moreover, it has been recently described that autophagy restricts the metastatic outgrowth in a mammary tumor model [129]. The inhibition of autophagy also induces the activation of survival pathways such as pro-inflammatory cytokine secretion [130], NRF2 [131], or alternative nutrient procurement pathways such as micropinocytosis [132]. These events indicate that targeting autophagy for cancer therapy depends on the tumor context and stage, and that a successful outcome is more likely to occur with a combined inhibition of multiple cellular pathways. In obesity-associated cancer, autophagy can be modulated by the adipokines secreted by the adipose tissue [133], which could be associated with metastasis, recurrence, and resistance to anticancer therapies in patients with obesity [3].

## 4. Drugs for Targeting Autophagy

Since autophagy has been involved in the pathogenesis of several diseases, research on drug development for targeting this process is an ongoing effort. Several drugs have been found to modulate the autophagic process, including clinically available drugs, new drugs used in pre-clinical studies, and many natural products targeting autophagy. In Table 1, we include some of the available autophagy modulators that are being used in pre-clinical and clinical studies for targeting autophagy in diverse diseases.

## 5. Autophagy Regulation by Adipokines in Cancer

**Figure 3:**
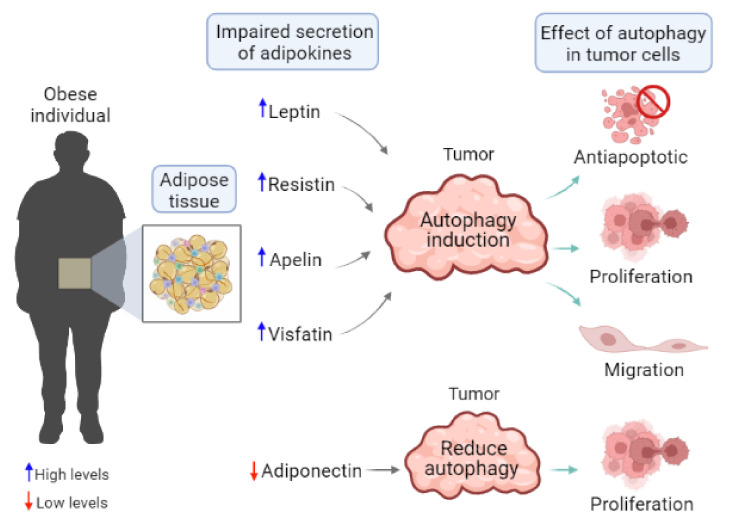
Adipokine-mediated autophagy on tumor cell characteristics during obesity. Adipokines such as leptin, resistin, apelin, and visfatin are elevated in obese individuals with cancer and have been related to a poor prognosis. It has been demonstrated that these adipokines induced autophagy in tumor cells. Adipokine-induced autophagy has been associated with pro-tumoral effects, such as the evasion of apoptosis, increased proliferation, and cell migration. On the other hand, adiponectin is the only adipokine present at low levels in an obese individual. The effect of low adiponectin conditions on autophagy is still being explored. However, in a breast cancer transgenic mouse model, it was demonstrated that whole-body adiponectin deficiency increased tumor growth. Tumor progression was attributed to a lack of autophagy induction and decreased autophagic degradation of LDL receptor in the absence of adiponectin. These results suggest an essential role for adiponectin deficiency in regulating lipid metabolism in obesity-associated malignancies through the regulation of autophagy.

Since the report that physiological levels of leptin could control autophagy in multiple peripheral tissues [143], there has been increased interest in describing the effects of other adipokines secreted during obesity on tumor cell autophagy and their effect on tumor progression (Figure 3).

Malik et al. (2011) were the first to report that exogenous leptin treatment induced autophagy in diverse mouse tissues and cancer cell lines (HeLa, HCT116, U2OS) by inducing AMPK phosphorylation and mTOR inactivation. However, their study was limited to demonstrating changes in autophagy, and did not evaluate the effects of leptin-mediated autophagy on characteristics related to tumor progression. One of the first reports on the role of adipokines in tumor cell autophagy and its effects on cancer-related features used U266 and ARP-1 multiple myeloma (MM) cells cultured with a conditioned medium from bone marrow-derived adipocytes [144]. In this work, the authors found that leptin and adipsin were the most abundant soluble factors in the adipocyte-derived conditioned medium, and were responsible for increasing autophagy in MM cells [144]. They also revealed that JAK2/STAT3 activation could be the main pathway of leptin- and adipsin-mediated autophagy induction, and that increased autophagy had an apoptosis-protective effect related to decreased PARP cleavage in MM cells (Table 2) [144].

**Figure 4:**
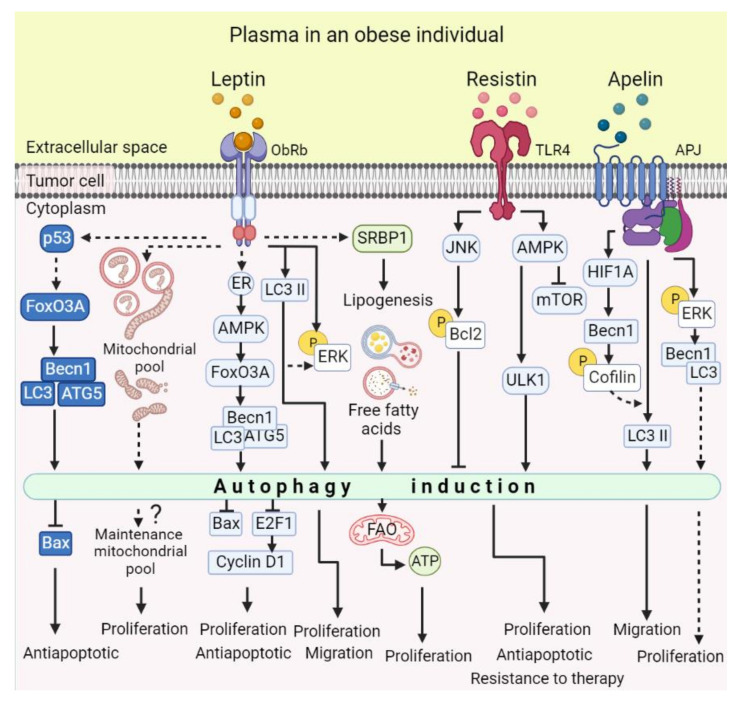
Molecular mechanisms of the effect of adipokines on autophagy and tumor cell malignancy. Different mechanisms involved in the induction of autophagy by leptin have been described. Leptin-induced autophagy has been shown to regulate proteins involved in proliferation and apoptosis, such as cyclin D1 and Bax. The autophagy-mediated regulation of Bax involves the p53/FoxO3A axis, which transcriptionally regulates autophagy-related proteins. Specifically, in ER-positive breast cancer cells, leptin-induced autophagy is ER-dependent, involves AMPK activation, and regulates Bax and Cyclin D1 levels. Leptin-induced autophagy is implicated in cellular migration through the regulation of ERK phosphorylation. Additionally, leptin-induced autophagy could be a mechanism for maintaining the mitochondrial pool, which would influence energetic metabolism. In this sense, leptin-induced autophagy is involved in the degradation of intracellular fat droplets, which provide free fatty acids that are oxidized and improve cancer cell metabolism. The first evidence for the role of resistin in autophagy demonstrated that resistin induces autophagy by decreasing the phosphorylation of mTOR and ULK1, and upregulating p-AMPK. Another important signaling molecule involved in resistin-induced autophagy was JNK kinase, which mediated the phosphorylation of Bcl-2. Bcl-2 phosphorylation is known to lead to its dissociation from Beclin1, a required step in the autophagy process. On the other hand, apelin/APJ increases autophagy and promotes *Becn1* expression via HIF1A. Interestingly, Beclin 1 regulates cofilin phosphorylation, which was important for apelin/autophagy-induced migration in lung adenocarcinoma. Additionally, it has been demonstrated that apelin-induced Becn1 and LC3 protein levels are dependent on the phosphorylation of ERK. This suggests an essential role for ERK in regulating apelin-induced autophagy proteins. Solid line: demonstrated mechanism; dashed lines: unknown mechanisms.

The relationship between leptin and autophagy has become important because of its effects on the promotion of malignancy. In a study performed in MCF-7 breast cancer cells and HepG2 hepatocellular carcinoma cells, it was shown that the addition of exogenous leptin increased the expression of autophagy-related genes such as Beclin-1, Atg5, and LC3, increased autophagosome formation, reduced the accumulation of p62 and increased autophagic flux (Table 2) [145]. Additionally, leptin increased p53 and FoxO3A protein levels, which were implicated in the induction of autophagy [145]. It was proposed that leptin induced autophagy through the activation of the p53/FoxO3A axis, and that autophagy was necessary for maintaining cellular proliferation and avoiding apoptosis by Bax suppression [145]. Additionally, Blanquer-Rosselló et al. (2015) observed that leptin increased the co-localization of lysosomes with mitochondria, suggesting an increase in mitophagy as a mechanism for maintaining the mitochondrial fraction in MCF-7 breast cancer cells (Figure 4) [38].

In breast cancer, the estrogen receptor (ER) is a determinant factor for leptin-mediated autophagy induction. ERα silencing reduced the leptin-induced expression of Atg6 and Beclin-1, as well as LC3 I to LC3 II conversion, and increased p62 protein levels. Additionally, leptin-induced phosphorylation of the autophagy positive regulator AMPK and increased FoxO3A protein levels were dependent on the presence of the ER (Figure 4) [146]. The authors propose that leptin induces autophagy via the ER-dependent AMPK-FoxO3A pathway [146]. The importance of leptin-induced autophagy in ER+ breast cancer cells relies on leptin-induced cancer cell proliferation, as autophagy was shown to participate in the negative regulation of the transcriptional factor E2F1, enabling leptin-mediated cyclin D1 expression (Table 2) [146]. In line with the above, our group reported that autophagy is essential in leptin-induced migration in ER+ and TN breast cancer models, as the pharmacological and genetic inhibition of autophagy significantly reduced leptin-induced cell migration (Table 2, Figure 4) [147]. Although the exact mechanism has not been defined, we observed that autophagy inhibition also reduced leptin-induced ERK phosphorylation in both breast cancer subtypes [147], probably suggesting ERK involvement in cell migration-associated events such as actin cytoskeleton rearrangement.

Interestingly, an important role for autophagy has been proposed in leptin-induced metabolic reprogramming. In estrogen receptor (ER)-positive breast cancer cells, leptin increased ATP generation by inducing a metabolic switch to fatty acid metabolism [148]. This switch involved an increase in the expression of fatty acid synthase (FAS) and other enzymes related to fatty acid synthesis, modulated by an increase in the transcription factor SREBP-1. These mechanisms increased the abundance of intracellular free fatty acids (FFAs) and increased fatty acid oxidation (FAO) [148]. These data demonstrated that leptin-induced autophagy is essential for increased SREBP-1 expression, leptin-induced fatty acid synthesis, and oxidation. The authors propose that leptin-induced autophagy allows the degradation of intracellular fat droplets de novo, and the subsequent increase and mobilization of FFAs to FAO. This metabolic switch would improve cancer cell metabolism and induce tumor growth [148]. The demonstration that leptin regulates cancer cell autophagy highlights the importance of autophagy in the anti-apoptotic, proliferative and migratory effects, and metabolic reprogramming, associated with leptin-mediated tumor progression (Figure 4).

**Table 2:** Effects of adipokines on autophagy in different cancer models. In several experimental models, it has been shown that the exogenous addition of adipokines induces autophagy and promotes cancer progression. Although adiponectin is found at low levels in an obese individual, it has been shown that exogenous adiponectin induces autophagy, which is a key mechanism for some antitumor effects induced by adiponectin.

Adipokines	Effect on Autophagy	Effect of Autophagy in Cancer	Experimental Model	Cancer Type	Reference
**Leptin**	Inductor	-	HeLa	Cervical	[143]
			HCT116	Colorectal	
			U2OS	Osteosarcoma	
**Leptin and** **adipsin**	Inductor	Protects against apoptosis	U266	Multiple Myeloma	[144]
			ARP-1		
			Tumor xenograft		
**Leptin**	Inductor	Maintains proliferation and prevents apoptosis	MCF-7	Breast	[145]
			HepG2	Hepatocellular carcinoma	
			Tumor Xenograft		
**Leptin**	Inductor	Promotes tumor growth and proliferation	MCF-7	Breast	[146]
			Tumor xenograft		
**Leptin**	Inductor	Promotes proliferation, migration, and morphological change	MCF-7	Breast	[147]
**Resistin**	Inductor	Inhibits doxorubicin-induced apoptosis	MCF-7	Breast	[149]
**Apelin**	Inductor	Promote proliferation and cell migration	A549	Human lung adenocarcinoma	[90]
**Adiponectin**	Inductor	Increased proliferation in glucose-deprivedmedium	DLD-1	Human Colorectal	[150]
			HT-29	Mouse Colorectal	
**Adiponectin**	Inductor	Adiponectin deficiency fails to induce autophagy, avoiding LDLR turnover and increasing tumor growth. Adiponectin treatment induces autophagy and rescues adiponectin deficiency effects.	Adiponectin deficient MMTV-PyMT mice and MDA-MB-231	Breast	[151]
**Adiponectin**	Inductor	Inhibits adiponectin- induced apoptosis	MCF-7	Breast	[152]
			HepG2	Hepatocellular carcinoma	
**Adiponectin**	Inductor	Cytotoxic, contributes to cancer cell death and decreases tumor growth	MCF-7	Breast	[153]
			MDA-MB-231		
			Tumor xenograft		

Only one study has evaluated the effect of resistin on autophagy in cancer cells. According to the observations of Liu et al. (2017), resistin stimulated autophagy in breast cancer cells through the activation of the AMPK/mTOR/ULK1 and JNK axis (Figure 4). Importantly, resistin-induced autophagy in MCF-7 and MDA-MB-231 cells inhibited apoptosis mediated by doxorubicin, a drug used in adjuvant chemotherapy and recommended as a first-line treatment for breast cancer (Table 2). This work underscores the association of autophagy with resistance to anticancer drugs, and suggests that elevated resistin levels in obese breast cancer patients could induce cancer cell chemoresistance through autophagy [149].

Apelin has been reported as an oncogene for breast, lung, prostate, gastric, ovarian cancer, etc. [154]. However, its relationship with autophagy has only been described in lung adenocarcinoma. Immunohistochemical analysis for detecting the apelin receptor, APJ, showed that tumors from patients with lung adenocarcinoma had high levels of APJ compared to adjacent tissue. Likewise, patients had elevated levels of apelin in plasma that were associated with poor prognosis [90], which led to the investigation of the protumoral mechanisms of apelin. In lung adenocarcinoma cell lines, it was shown that apelin-13 promotes proliferation through the expression of cyclin D1 mediated by the phosphorylation of ERK1/2 (Table 2, Figure 4) [90]. Importantly, apelin induced autophagy with the possible participation of ERK signaling [90]. Furthermore, a high expression of autophagy-related genes in lung adenocarcinoma was identified, and it was shown that apelin-induced autophagy is required for lung adenocarcinoma cell migration, where cofilin phosphorylation was necessary for autophagy induction mediated by apelin [155]. Summarizing these findings, the authors proposed that combining autophagy and cofilin inhibitors could be a strategy against lung cancer metastasis, and that apelin could be a potential clinical prognostic marker associated with autophagy activation [155].

The role of visfatin/Nampt in autophagy and cancer has been described in the context of its enzymatic activity, and not properly as a circulating adipokine with tumor effects. It has been observed that cancer cells overexpressing visfatin/Nampt exhibit a significant dependence on NAD+ to support their rapid cell proliferation. In this way, the inhibition of visfatin/Nampt has been proposed to reduce tumor proliferation and growth. Some reports have described that visfatin/Nampt inhibitors have a cytotoxic effect associated with the induction of autophagy in vitro in multiple myeloma [156], osteosarcoma [157], T-cell leukemia [139], and glioblastoma [158,159]. It has been reported that the use of the chemical inhibitor of visfatin/Nampt, FK866 (also called APO866 or WK175), induces autophagy associated with cytotoxicity, leading to p53-independent cell death [157]. However, the effect of exogenous visfatin/Nampt on tumor autophagy has not been explored.

**Figure 5:**
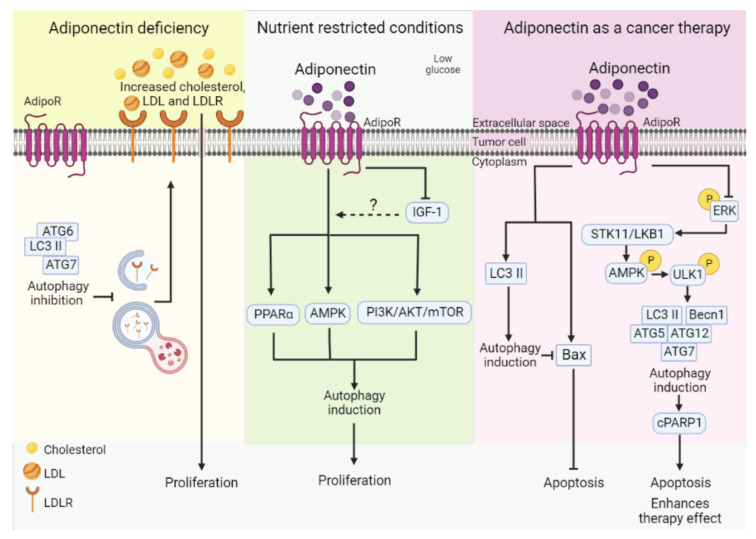
Regulatory mechanism of autophagy as determined by adiponectin and its different effects on cancer cell malignancy. Adiponectin deficiency reduces autophagy-related protein expression in primary tumor cells. A reduction in autophagy explains the increased cholesterol, LDL, and LDLR associated with accelerated tumor development in adiponectin deficiency conditions. This mechanism highlights autophagy as a possible pathway by which adiponectin deficiency might favor obesity-associated malignancies, especially breast cancer. On the other hand, supplying adiponectin has been proposed as a cancer therapy, particularly in obesity-associated cancer, since the addition of high levels of adiponectin to tumor cells induced autophagy and apoptosis. In this sense, it has been shown that adiponectin-induced autophagy negatively regulates apoptosis via the downregulation of Bax. Because autophagy inhibits adiponectin-induced apoptosis, it was suggested that the inhibition of autophagy would be an important therapeutic approach for enhancing the efficacy of cancer treatment with adiponectin. Additionally, it has been demonstrated that adiponectin-induced autophagy contributes to adiponectin-induced cancer cell death. Adiponectin inhibits ERK phosphorylation, which negatively regulates STK11/LKB1-AMPK activation. STK11/LKB1 leads to AMPK activation, which in turn increases ULK phosphorylation and induces autophagy. Adiponectin-induced autophagy was associated with the cleavage of PARP1 driven by adiponectin.

Different studies show that adiponectin has an autophagy-promoting effect (Table 2, Figure 5). However, the effect of adiponectin-induced autophagy on cancer cells varies according to experimental and metabolic conditions, or the stage of tumor progression. As mentioned previously, adiponectin is the only adipokine with beneficial health effects due to its role in the maintenance of lipid metabolism and glucose homeostasis. During obesity, adiponectin levels decrease, and this might be related to the effects of obesity on increasing cancer risk [11]. One study has shown a tumor-suppressor role for adiponectin-induced autophagy, implicating autophagy in the cancer repressive effects mediated by adiponectin. In the MMTV-PyMT transgenic mouse model, the genetic deficiency of adiponectin elevated plasma cholesterol and low-density lipoprotein (LDL) levels, as well as increased low-density lipoprotein receptor (LDLR) expression, contributing to breast cancer development. Autophagy markers LC3 II, Atg7 and Atg6 were reduced in primary tumor cells (Figure 5; Table 2) [151]. Interestingly, in breast cancer MDA-MB-231 cells, exogenous adiponectin decreased LDL cholesterol-induced proliferation, cholesterol uptake, LDL levels and LDLR, and increased autophagy markers [151]. The authors propose that autophagy contributes to LDLR turnover by degrading it in the lysosomes, and thus autophagy inhibition by adiponectin deficiency decreased the receptor turnover and enhanced lipid signaling, promoting cancer cell proliferation [151]. This study positions autophagy as an essential regulator of lipid homeostasis and a critical mediator of the anti-proliferative and tumor suppressive effect of adiponectin.

On the contrary, high adiponectin levels have been linked to a reduction in cancer development and progression [11], and adiponectin has been suggested as a strategy for the treatment of many obesity-related malignancies. In this setting, the effects of adiponectin-induced autophagy on cancer cells vary depending on the experimental setting. Accordingly, in human DLD-1 and mouse HT-29 colorectal cancer cell lines, both the full-length and globular isoforms of adiponectin reduced proliferation in complete medium and increased proliferation under low glucose conditions [150]. The proliferative effect of adiponectin under low glucose conditions was associated with increased autophagy, which was activated as a consequence of the inhibitory effect of adiponectin on IGF-1, allowing the activation of AMPK and PPAR and the subsequent inhibition of PI3K/AKT/mTOR (Table 2 and Figure 5) [150]. This study suggests that adiponectin may have supportive effects on tumor cell survival through autophagy induction, considering that tumor cells are generally under nutritional stress [150].

On the other hand, Park et al. (2014) demonstrated that globular adiponectin induced autophagy and apoptosis in HepG2 hepatocellular carcinoma and MCF-7 breast cancer cells. Interestingly, the inhibition of autophagy enhanced caspase activity and Bax protein levels in both cancer cell lines (Figure 5) [152]. These findings suggest that autophagy negatively regulated adiponectin-induced apoptosis, and that the inhibition of autophagy might function as a therapeutic approach for enhancing the efficacy of adiponectin-mediated cancer treatment. 

In contrast to the previous findings, Chung et al. (2017) reported that adiponectin treatment decreased breast cancer cell proliferation and tumor growth with the induction of autophagy induced by the activation of the STK11/LKB1-AMPK-ULK1 axis (Figure 5). In this study, autophagy contributed to cancer cell death, since its inhibition restored breast cancer cell survival and proliferation [153]. Altogether, these works on adiponectin-induced autophagy demonstrate that autophagy can negatively modulate apoptosis, increase proliferation under low glucose conditions, participate in the promotion of cell death or participate in the tumor-suppressive functions of adiponectin. Due to the dual role of autophagy in cancer, depending on the tumor stage, it is expected that adiponectin-induced autophagy will have different roles depending on the stage of cancer progression. Moreover, since adiponectin directly regulates the PI3K/AKT and AMPK signaling pathways, as well as lipid and glucose metabolism [11], the metabolic context of the cancer cell might affect the role of autophagy. So, it will be necessary to explore the role of autophagy induced by adiponectin in different experimental settings, and evaluate different stages of cancer progression, to identify whether adiponectin in combination with the inhibition of autophagy could be implemented in the therapy of obesity-associated cancers.

## 6. Perspectives and Limitations of Targeting Adipokine-Induced Autophagy for Cancer Treatment

Despite being considered a satiety hormone, leptin, and other adipokines have been shown to induce autophagy in cancer cells. This effect might sound counterintuitive since autophagy is considered a cell survival pathway activated under different types of cellular stress, particularly nutritional stress, or starvation. However, studies have reported that autophagy in adipose tissue correlates with the degree of adiposity in patients and obese mice, and autophagic activity has also been shown to positively correlate with adipose tissue inflammation [106]. Thus, increased autophagy in cells exposed to exacerbated adipose tissue-mediated signaling, as happens in obese individuals, occurs in conditions of nutrient abundance. 

However, inducing autophagy in cancer cells has been shown to not only help cancer cells survive starvation, but to have significant roles during the metastatic process (Figure 3). For example, autophagy can induce focal adhesion turnover to facilitate cellular movement; it can promote anoikis resistance to enable cellular survival upon extracellular matrix detachment, and can support cancer stem cell (CSC) survival [160,161]. On the other hand, autophagy has also been shown to negatively regulate metastasis, since it degrades EMT-related transcription factors and prevents inflammation [160,161]. Since leptin-induced autophagy has been shown to induce cancer cell migration, it will be essential to outline the cellular mechanisms involved. This could help in identifying and explaining the conditions in which autophagy exhibits metastasis-promoting versus metastasis-inhibiting functions. It will also be crucial to define if different adipokines promote autophagy by activating similar or different pathways to those leptin does—particularly adiponectin, which is elevated in normal-weight individuals, and where, in the absence of obesity, autophagy would be expected to display its tumor-suppressive function.

Another important question regarding the role of adipokine-mediated signaling in autophagy is whether leptin and other adipokines have a similar function in transformed and non-transformed cells. In this regard, it has been shown that chronic leptin treatment induces EMT in a non-tumorigenic cell line [162]. Still, leptin-induced autophagy in the regulation of EMT has not been investigated in this model. Additionally, a recent study described that autophagy in differentiating adipocytes is important for the differentiation process and the regulation of adipokine production, indicating that autophagy in adipocytes also contributes to the maintenance of adipose tissue and adipokine secretion with essential functions in the maintenance of cancer cell proliferation and migration [163].

Autophagy also has a crucial role in regulating energy metabolism, where it maintains energy balance upon nutrient deficiency [106]. However, in adipokine signaling, leptin has been shown to promote fatty acid oxidation [32], and autophagy has been implicated in promoting this metabolic shift [148] by modulating lipophagy, or the lysosomal degradation of lipid droplets. This might be an interesting metabolic switch that allows cancer cells to utilize the metabolic fuel that is the most available in obese individuals. It will be interesting to understand how shifting the primary energy source to lipids contributes to leptin-induced proliferation and invasion. 

Autophagy has also been implicated in both white and brown adipocyte differentiation; autophagy inhibition has been shown to increase proinflammatory cytokine expression in the adipose tissue of obese mice or patients with obesity [106] and obesity affects autophagy in the adipose tissue [164]. Moreover, upon starvation, autophagy modulates the secretion of acyl-CoA binding protein (ACBP), which has multiple effects on metabolism, including increasing glucose transport to hepatocytes and adipose tissue, inhibiting fatty acid oxidation, and inducing a hyperphagic response due to the activation of orexigenic neurons in the hypothalamus [165]. Thus, starvation-induced autophagy regulates the hyperphagic response to starvation, promoting lipogenesis and obesity. Importantly, although ACBP is not considered an adipokine since it can be secreted by several tissues, it has been compared to leptin, since its levels are high in obesity, but it shows an association with high levels of obesity with its effects as an orexigenic factor [165,166]. Thus, autophagy has important functions in the maintenance of metabolism, response to nutritional stress and adipose tissue differentiation, which are likely to be disrupted in obesity, and it will be imperative to clearly identify differences in autophagy and its effects under normal and obese conditions if we want to target this process in obese patients.

Finally, autophagy has also been shown to have metastasis-inhibiting functions in advanced disease models. For instance, autophagy in disseminating tumor cells degrades NBR1, a protein required for metastatic outgrowth and basal differentiation. In this setting, autophagy inhibition in metastatic cells in mouse models of breast cancer increased metastatic size, number and basal differentiation, which is associated with increased metastasis [129]. Additionally, autophagy has been implicated in autophagy-dependent cell death, particularly in cells with defects in the apoptotic pathway or when specific death pathways are activated [167]. These findings indicate that caution should be taken when trying to manipulate autophagy in the context of cancer in obese patients, and to carefully analyze possible side effects. Thus, it will be important to clearly understand the role of autophagy in the modulation of cancer to effectively target this process for cancer therapy. Furthermore, since autophagy has such an important role in the diverse mechanisms related to cancer progression, it will be important to clearly understand the mechanistic insights related to adipokine-mediated signaling in the regulation of autophagy and cancer-promoting effects in order to fully delineate the interplay between adipose tissue and its cancer-promoting effects.

## 7. Conclusions

The spread of obesity has led to its recognition as an ongoing pandemic [1]. Its relationship with the risk of developing several diseases, including cancer, has emphasized the need to improve obesity prevention and treatment strategies. In cancer patients, adipose tissue is known to have important effects on cancer cell signaling, mostly promoting tumor progression. Since adipokines are among the main bioactive secreted factors in adipose tissue, it is important to understand their specific effects on cancer cells. Autophagy is one of the mechanisms implicated in tumor suppression and progression, and evidence indicates that adipokines induce autophagy. Thus, inhibiting this process is an interesting target in cancer with obesity. It will be important to clearly delineate the effects of adipokines on autophagy and their outcome on diverse cancer cells from different cancer stages. Since autophagy is relatively easy to target in patients, it might be a promising therapeutic strategy for cancer patients with obesity.

## Figures and Tables

**Table 1:** Pharmacological and natural products known to modulate autophagy.

Autophagy Inducer/ Inhibitor	Target	Drug(s)	Reference(s)
**Clinically available** **inducers**	mTOR inhibitors	Rapamycin	[134]
		Everolimus	
		Temsirolimus	
	PI3K/mTOR inhibitors	Dactolisib	
	AMPK activators	Metformin	
		Simvastatin	
	Lipid metabolism	Carbamazepine	
**Inducers with potential** **clinical relevance**	Sirtuins	Polyphenols	[135]
		(resveratrol)	
	Unknown	Phenolic oleosides	[135,136,137,138]
		Caffeine	
		Alkaloids	
		Terpenes and terpenoids	
		Ilimaquinone	
		Paratocarpin E	
	Beclin 1/Vps34	Tat-beclin-1	[139]
	TFEB	Trehalose	[135,140]
		Curcumin	
	AMPK activators	Polyphenols	[140]
		Epigallocatechin gallate	
		Kaempferol	
		Quercetin	
**Clinically available** **inhibitors**	Lysosomotropic agents (antimalarials)	Chloroquine	[134]
		Hydroxychloroquine	
**Inhibitors with potential clinical relevance**	Lysosomotropic agents	Lys05	[141]
	Vps34 inhibitors	3-MA	[134]
		Wortmannin	
	ULK1/2 inhibitor	SBI-0206965	
		MRT67307	
		MRT68921	
	USP13 and USP10 inhibitor	Spautin-1	
	ATG4B inhibitor	NSC185058	
	Microtubules	Vinca alkaloids	[140]
		Colchicine	
	Autophagosomal acidification	Azithromycin	
		Clarithromycin	
		Matrine	
	Unknown	Lucanthone	[140,142]
		Coibamide A	
		Dicitrinone B	
	ATP synthase inhibitor	Bafilomycin A	[141]
	P62	Verteporfin	[134]
